# Management of an Anticipated Difficult Airway in a Pediatric Patient With Stickler Syndrome

**DOI:** 10.7759/cureus.49622

**Published:** 2023-11-29

**Authors:** Joana Veiga, Pedro Moreira, Elsa Soares, Helena Salgado

**Affiliations:** 1 Anesthesiology, Hospital de Braga, Braga, PRT; 2 Anesthesiology, Hospital Santa Maria Maior, Barcelos, PRT

**Keywords:** stickler syndrome, pierre robin sequence, fiberoptic intubation, difficult airway management, upper airway ultrasound

## Abstract

Stickler syndrome (SS) is a congenital autosomal dominant condition that affects the formation of collagen. Of primary importance to the anesthesia provider is the propensity for difficulties in managing the airway due to orofacial abnormalities associated with this syndrome.

The authors present a two-year-old infant with SS who required anesthetic care for a cleft palate repair. The potential anesthetic implications of this syndrome are discussed and the importance of proper planning and preparation and the usefulness of ultrasound as an airway evaluation tool are highlighted.

## Introduction

Stickler syndrome (SS) is an autosomal dominant collagenopathy, with a reported prevalence of one in 7,500-9,000 newborns [1]. Consequences of defective collagen molecules or reduced amounts of collagen affect the development of bones and other connective tissues, leading to the characteristic features of SS. This genetic disease can range from mild, in which there are no manifestations of the syndrome, to severe, in which all organ system is involved. SS has a broad spectrum of craniofacial manifestations, that also include malar hypoplasia, a small jaw, high arched palate, a broad nasal bridge or micro/retrognathia, and a propensity for upper airway obstruction [2]. The prevalence of cleft palate in SS patients is estimated at approximately 40%, and 24% of patients are born with Pierre-Robin Sequence (small lower jaw, cleft palate, and tongue-bases obstruction) [2,3], a condition known to predispose patients to perioperative airway and respiratory complications [4]. Anesthetic management reports of SS patients are scarce but described complications include difficult facemask ventilation, oxygenation, and/or intubation, which may therefore represent an anticipated difficult airway (ADA) scenario.

While current literature suggests that airway management in SS may be a concern, reports on how to manage these difficult airways are rare, special in the pediatric population. To improve knowledge of such a quite rare disease and to improve safety in everyday practice, we believe this report is an asset, especially for pediatric surgical centers with less experience in genetic disorders. Here, we highlight the anesthetic challenges faced by our anesthetic team in a pediatric patient with SS scheduled for elective surgery and the usefulness of ultrasound as an airway evaluation tool.

## Case presentation

A two-year-old male, weighing 14 kg, exhibiting cranial features of Pierre-Robin sequence and genetic confirmation compatible to SS (COL11A1 gene mutation, variant c.2952+1G>T), presented for cleft palate repair in our tertiary care hospital. He had a positive history of roncopathy due to tongue-based obstruction, malar hypoplasia, microcephalia, and low-set ears. Airway evaluation revealed a small mouth opening, thyromental distance of <2cm, a small lower jaw, retrognathia, and Mallampati Class III. He had one previous anesthesia record for the same procedure one year before, presenting with similar craniofacial features and weighing at that time 9.7 kg. During that anesthetic procedure airway intubation was attempted with videolaryngoscopy, while maintaining spontaneous ventilation, using CMAC® with Miller 1 blade and Macintosh (Mac) 2 blade, and in both cases, a Cormack-Lehane (CL) grade 4 was encountered, and no attempt of intubation was made. In this previous episode, intubation was achieved with fibroscopy through an endoscopic face mask and a standard cuffed 4.0mm endotracheal tube (ETT) was placed. Without the availability of a reinforced 4.0mm ETT (not available at that time at the hospital-reinforced ETTs <4.5mm), surgery could not proceed, as the placement of the mouth gag precluded adequate ventilation. The child was wakened, and surgery was postponed.

For the current procedure, the anesthetic team learned and anticipated the difficulties previously felt and requested in advanced reinforced ETTs 4.0mm and checked the availability of adequate fiberscope. Intranasal dexmedetomidine premedication (1 mcg/kg) was administered to maintain the infant less agitated during pre-oxygenation and until venous access was obtained and nebulization with dexamethasone (0.3 mg/kg) and lidocaine (1.5 mg/kg) was administered to diminish airway reactivity during the procedure.

**Figure 1:**
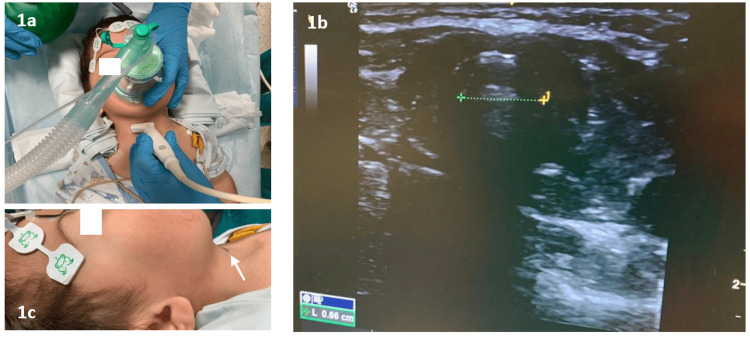
(a) Airway ultrasound after inhalation anesthesia with Sevoflurane, using a “hockey stick” probe (23 mm) to measure the tracheal diameter. (b) Ultrasound image of tracheal diameter measuring. (c) Arrow indicating cricotiroid membrane ultrasound-guided mark in the neck.

ASA standard monitoring plus cerebral oximetry, depth of anesthesia with bispectral index monitoring, and muscle relaxation monitoring with Train-Of-Four (TOF) were used. After inhalational anesthesia with sevoflurane, with low MAC to maintain spontaneous ventilation, we performed airway ultrasound using a linear “hockey stick” probe (23 mm) to measure the tracheal diameter (6.6 mm), to choose the correct size of reinforced ETT to be used, and marked the cricothyroid membrane, in the unlikely needed of an emergency front of neck access (eFONA) (Figures 1a-1c). Also, an ENT surgeon was readily available.

**Figure 2:**
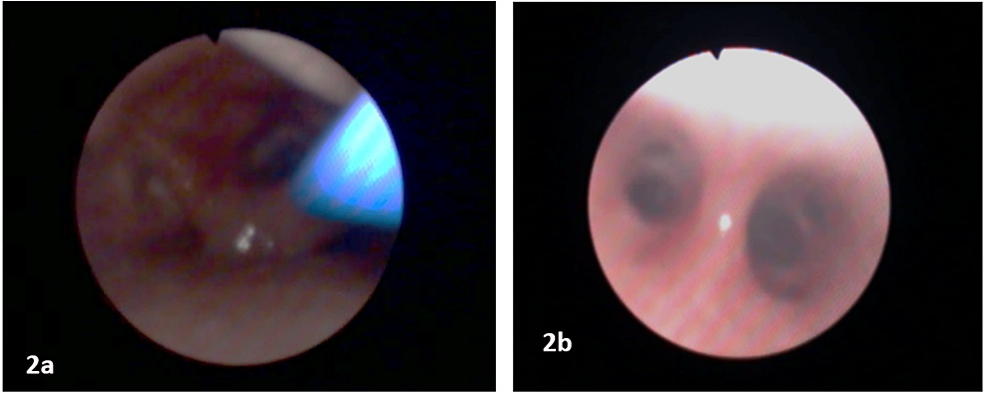
(a) Fibroscopy image of vocal cords and spray as you go with lidocaine through the epidural catheter to reduce airway hyperreactivity. (b) Carina, the landmark to properly place the orotracheal tube.

Carefully preserving spontaneous breathing, a fiberoptic assisted tracheal intubation (Karl Storz® 2.2 mm), through an endoscopic facemask to maintain inhalation anesthesia and oxygenation, was accomplished with a reinforced cuffed 4.0 mm ETT. Airway reactivity was further blunted by complementing the nebulization using the spray-as-you-go technique with lidocaine (another 1.5mg/kg in total) through an epidural catheter in the fiberscope working channel (Figures 2a, 2b).

After visualization of the ETT within the tracheal lumen and capnography confirmed, we proceeded to induction with propofol (3mg/kg) and rocuronium (0.6mg/kg), and we maintained anesthesia with Sevoflurane. After intubation, videolaryngoscopy with C-MAC® Mac 2 Blade was performed, and still, a CL grade 4 was registered. The surgical procedure and anesthetic emergency were uneventful. The patient was then admitted to a Pediatric High Dependency Unit for post-operative vigilance.

## Discussion

This case aims to describe our anesthetic management, in particular the airway approach, of an infant with the typical phenotype of SS. Also, we highlight the utility of airway ultrasound and flexible fiberscope in clinical practice when orotracheal intubation is mandatory and we have to deal with a pediatric ADA, due to craniofacial findings typical for SS, including microcephaly and Pierre-Robin Sequence.

Point-of-care ultrasonography (POCUS) of the airway is becoming a first-line noninvasive adjunct assessment tool of the pediatric airway [5]. Successful use requires a thorough understanding of airway anatomy and ultrasound experience and may provide the anesthesiologist with valuable and personalized information about the patient's airway - identification of vocal cord dysfunction and pathology, assessment of airway size, prediction of the appropriate diameter of endotracheal and tracheostomy tubes, differentiation between tracheal and esophageal intubation, localization the cricothyroid membrane for emergency airway and identification tracheal rings for US-guided tracheostomy. In this case report and in scenarios like this one, where tracheal intubation is necessary and there is an ADA, the previous ultrasound examination of the airway allowed us to choose the appropriate size of the tracheal tube, especially important when using reinforced ETTs whose outer diameter is larger, and thus increasing the success rate of intubating the child on the first attempt. In addition, with the use of ultrasound, we were also able to accurately localize the cricothyroid membrane before induction, a simple clinical skill that can be very useful in the case of eFONA. The major disadvantage of ultrasonography remains interobserver variability, and operator dependence, as it requires specific training and experience. Current consensus and guidelines alert us to the role of airway ultrasound. Although it is not standard of care yet, there is significant potential for the integration of ultrasound technology into the routine care of the airway. With this case, we provide further positive arguments about the importance of airway ultrasound in the preparation and approach to a difficult airway.

Fiberoptic-assisted tracheal intubation remains a useful tool in the management of ADA in children in elective procedures and is a strategy in all ADA guidelines [6]. Although awake fiberoptic endotracheal intubation is commonly performed in adults, sedation or general anesthesia is frequently required in pediatric patients. Zimmerman et al. [7] reported a case series of 502 anesthetic events in patients with SS; in this article, fibroscopy was used for approaching the airway in only two cases, showing that this option is not the most used, even in ADA scenarios. The reason behind the low use may be the absence of this material, the lack of competent people to use it, or the ease of other gadgets such as videolaryngoscopes. In our case, videolaryngoscopy had proven not to be useful, and fiberoptic-assisted tracheal intubation was our first line option, complemented with previous airway POCUS, and preparation for a possible event of eFONA was considered.

During the performance of fibroscopy in children with ADA, it is extremely important to maintain spontaneous ventilation, even during sedation or general anesthesia in the case of a non-cooperative child. Although we used inhalational anesthetic agents to achieve that, given its limited impact on respiratory function, dexmedetomidine also seems to be a good sedative agent in similar cases [8]. Supraglottic airway device was not a first line option here, since the procedure required an orotracheal tube to maintain safety and provide space for the surgeon to work, it still could be useful as a rescue device or intubation guidance.

This case report highlights how adequate preparation is key for success, especially in suspected or ADAs in children. Also, it shows one of the still little-explored potentials of airway ultrasound as an extremely useful tool for pre-operative airway evaluation in children. Lastly, this clinical case reminds us that not always videolaryngoscopy saves the day - maintaining skills if fiberoptic intubation is essential.

## Conclusions

This case report describes the usefulness of airway ultrasound and fibroscopy in managing an ADA in pediatric patients. In challenging cases as the case reported, an airway ultrasound evaluation to ensure our choice for an orotraqueal size tube and to landmark cricothyroid membrane in case of EFONA may be considered. Also, fibroscopy should not be devalued even in cases of pediatric difficult airway, because it allows orotracheal intubation in spontaneous breathing in case of an ADA.
